# The causal effect of two occupational factors on osteoarthritis and rheumatoid arthritis: a Mendelian randomization study

**DOI:** 10.3389/fpubh.2023.1281214

**Published:** 2024-02-12

**Authors:** Jian Huang

**Affiliations:** Clinical Laboratory Center, The First Affiliated Hospital of Guangxi Medical University, Nanning, China

**Keywords:** Mendelian randomization, osteoarthritis, rheumatoid arthritis, occupational factor, causal effect

## Abstract

**Background:**

Osteoarthritis (OA) and rheumatoid arthritis (RA) are two common types of arthritis. We conducted a two-sample Mendelian randomization (MR) study to estimate the causal effects of two common occupational factors—job involves heavy manual or physical work and job involves mainly walking or standing—on OA and RA in individuals of European ancestry.

**Methods:**

Instruments were chosen from genome-wide association studies (GWASs) that identified independent single nucleotide polymorphisms (SNPs) robustly linked to job involves heavy manual or physical work (*N* = 263,615) as well as job involves mainly walking or standing (*N* = 263,556). Summary statistics for OA and RA were taken from the Integrative Epidemiology Unit (IEU) GWAS database; both discovery and replication GWAS datasets were considered. The primary analysis utilized the inverse variance weighted (IVW) MR method supplemented by various sensitivity MR analyses.

**Results:**

In the IVW model, we found that genetically predicted job involves heavy manual or physical work was significantly associated with OA in both the discovery [odds ratio (OR) = 1.034, 95% confidence interval (CI): 1.016–1.053, *P* = 2.257 × 10^−4^] and replication (OR = 1.857, 95% CI: 1.223–2.822, *P* = 0.004) analyses. The causal associations were supported in diverse sensitivity analyses. MR analyses suggested no causal effect of genetically predicted job involves heavy manual or physical work on RA. Similarly, our data provided no evidence that genetically predicted job involves mainly walking or standing was related to OA and RA.

**Conclusions:**

Our MR study suggests that job involves heavy manual or physical work is a risk factor for OA. It is of utmost importance to create preventive strategies aimed at reducing its impact on OA at such work sites.

## Introduction

Arthritis is a prevalent condition that leads to joint pain and inflammation. It is estimated that arthritis affects more than 200 million people worldwide (1). The most common types of arthritis are osteoarthritis (OA) and rheumatoid arthritis (RA) (1). OA, also known as degenerative arthritis, is characterized by synovial inflammation, the breakdown of joint cartilage, chronic pain, and functional impairment. In Europe, approximately 40 million people suffer from OA; the estimated annual cost related to OA can reach €817 billion (2). RA, on the other hand, is a chronic inflammatory disorder that can affect multiple joints in the body. It is characterized by morning stiffness, symmetrical and erosive synovitis, production of autoantibodies, and progressive joint damage (3). Each year, approximately 2.3 million people in Europe are diagnosed with RA (4). The estimated annual cost of RA in Europe amounts to €45.3 billion (4). Due to their significant burden, the prevention of OA and RA presents a major challenge in public health.

Observational studies have long observed associations between occupational factors and arthritis, although the results remain inconsistent. For example, many researchers showed that jobs involving heavy manual or physical work, such as long-term heavy lifting work and carrying heavy weights, might be important risk factors for developing OA (5–10). A few observational studies also revealed a positive association between heavy physical work and RA development (11, 12). However, these observational studies had some limitations. Specifically, under a cross-sectional design, most studies depended on subjects reporting their occupation status retrospectively, which greatly increased the likelihood of recall bias (8). In addition, the presence of OA or RA symptoms may affect subjects' occupation selection or physical task, thereby introducing bias (5). Furthermore, one key limitation of observational investigations is the inability to assess causality. Therefore, it is necessary to use effective and complementary approaches to investigate the relationship between occupational factors and OA and RA.

Mendelian randomization (MR) is a statistical method utilizing genetic variations as instruments to infer the causal impact of an exposure. The method has two advantages: it reduces confounding and diminishes reverse causality as genetic variants are randomly assigned at conception and cannot be altered by the disease's development and progression (13, 14). In the present study, we conducted MR to estimate the causal effects of two common occupational factors, including job involves heavy manual or physical work and job involves mainly walking or standing on OA and RA. Our efforts focus on identifying specific workplace activities that may be related to the risk of OA and RA. By using the MR technique, our study can provide a more robust assessment of the causal relationships, offering valuable information for public health interventions targeting occupational risk factors. Moreover, our study may help healthcare providers and policymakers develop targeted strategies to promote healthy work environments and reduce the risk of developing these debilitating conditions.

## Methods and materials

### Overview

**Figure 1:**
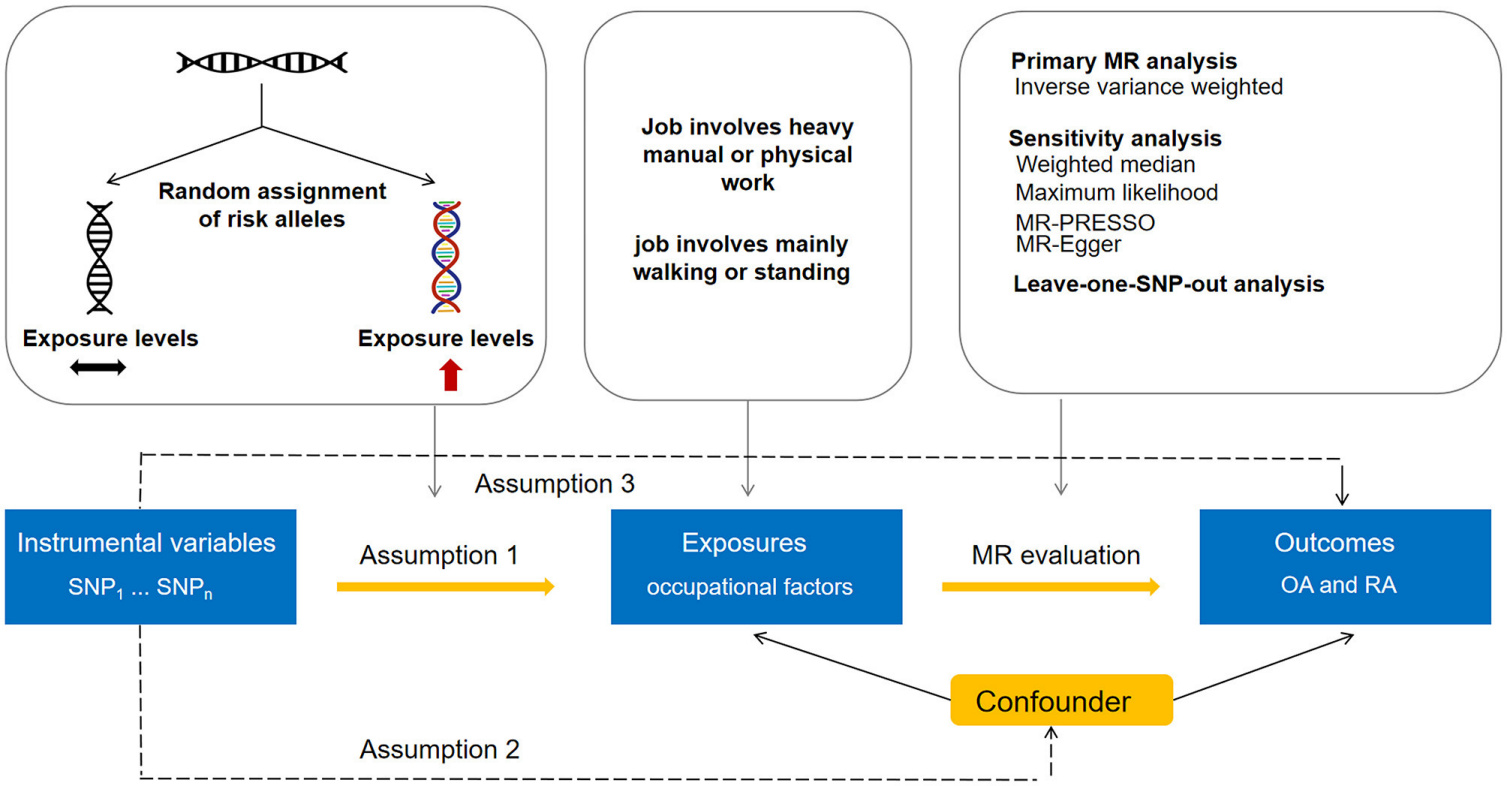
Overview of the Mendelian randomization analysis design.

The present two-sample MR study employed summary data obtained from publicly accessible genome-wide association studies (GWASs). Utilizing a two-sample design can enhance study power in that it allows for the incorporation of larger sample sizes (13). For each exposure, we estimated its association with OA or RA in a large discovery dataset, which was then followed by replicating the analysis using a second dataset. All GWASs acquired appropriate consent from participants and obtained ethical approval. Our study design is shown in Figure 1. Our analytic process adhered to the STROBE-MR guidelines (Supplementary Table 1).

### Data for exposures

For the MR analysis, genetic association estimates for job involves heavy manual or physical work were taken from a GWAS dataset (GWAS identifier: ukb-b-2002) constructed by the Medical Research Council-Integrative Epidemiology Unit (MRC-IEU) consortium including 263,615 European participants. Genetic variants for job involves mainly walking or standing were retrieved from another MRC-IEU GWAS dataset (GWAS identifier: ukb-b-4461) involving 263,556 European individuals. In these GWASs, the adjustment process considered 10 principal components of genetic ancestry, as well as age and sex. Based on data from the UK Biobank, a touchscreen questionnaire was used to obtain the participants' information on occupational factors. For job involves heavy manual or physical work, the participants were asked, “Does your work involve heavy manual or physical work?” This included work that involved the handling of heavy objects and the use of heavy tools. If the participants had more than one “current job,” they should answer this question for their main job only. Regarding job involves mainly walking or standing, the participants were asked the question: “Does your work involve walking or standing for most of the time?”

### Data for outcomes

Summary-level data for the associations of exposure-associated SNPs with OA were taken from a GWAS (GWAS identifier: ukb-a-106) by the Neale Lab involving 28,257 OA patients and 308,902 controls (discovery analysis). Additionally, data from another GWAS (GWAS identifier: ebi-a-GCST005811) were utilized, including 12,658 patients with OA and 50,898 controls (replication analysis) (15). In these GWAS, OA cases were defined based on self-reported information. The controls were those who did not self-report OA. Summary statistics for RA were selected from a GWAS (GWAS identifier: ukb-d-M13_RHEUMA) by the Neale Lab involving 1,605 RA cases and 359,589 controls (discovery dataset). We also took summary data on RA from another GWAS dataset (GWAS identifier: ebi-a-GCST90013534) by Ha et al. (16), involving 14,361 patients with RA and 43,923 controls for the replication analysis. RA patients were defined according to the 1987 American College of Rheumatology (ACR) criteria or were diagnosed by rheumatologists. The controls were individuals without RA (16). For reducing bias from population stratification, we selected all patients and controls of European ancestry.

### Instrumental variable selection

We selected SNPs that displayed a significant association with each exposure in the respective data source GWAS, reaching genome-wide significance (*P* < 5 × 10^−8^), to serve as instruments for the exposure. To ensure the independence of genetic variants, a window size of 10 Mb and a maximum linkage disequilibrium of *r*^2^ = 0.001 were used to clump the instruments. The selection of SNPs and clumping were done utilizing the TwoSampleMR package in R software (version 4.1.3) (17). We obtained 25 genome-wide significant SNPs for job involves heavy manual or physical work and 16 genome-wide significant SNPs for job involves mainly walking or standing. Tables 1, 2 show the characteristics of the instruments, including effect size (beta), standard error (SE), *p*-value, effect allele, other allele, and minor allele frequency. The F-statistics for the instruments in both job involves heavy manual or physical work (ranging from 22 to 121) and job involves mainly walking or standing (ranging from 28 to 107) surpassed 10, indicating enough strength for MR analyses.

**Table 1:** Significant SNPs associated with job involves heavy manual or physical work (*r*^2^ < 0.001).

**SNP**	**Position**	**Chromosome**	**EA**	**OA**	**EAF**	**Beta**	**SE**	***P***
rs2091329	110042079	1	G	A	0.288	−0.016	0.003	2.00E-09
rs12089815	91189933	1	A	G	0.549	−0.015	0.002	3.10E-10
rs2819348	201884952	1	C	T	0.352	0.015	0.002	5.60E-09
rs6544763	44813233	2	C	T	0.660	0.014	0.003	3.20E-08
rs11678979	100802891	2	C	T	0.272	−0.015	0.003	3.00E-08
rs34700731	203360745	2	A	G	0.135	0.020	0.003	1.50E-08
rs11130889	62562748	3	G	C	0.075	−0.025	0.005	4.50E-08
rs35999162	49597230	3	G	C	0.310	−0.025	0.003	7.40E-22
rs2318540	67831581	4	G	T	0.572	−0.014	0.002	3.70E-09
rs11726786	106120756	4	G	T	0.366	0.017	0.002	2.40E-11
rs1081158	88004616	5	T	C	0.582	0.017	0.002	2.70E-12
rs56194430	67824690	5	T	C	0.169	0.019	0.003	4.50E-09
rs4580876	98322872	6	A	G	0.476	−0.022	0.002	3.00E-20
rs11756123	152218079	6	T	A	0.635	−0.014	0.002	3.20E-08
rs4731992	133702097	7	G	A	0.782	−0.017	0.003	4.00E-09
rs13250996	13957985	8	C	G	0.504	−0.013	0.002	2.70E-08
rs77823953	143524185	8	T	C	0.033	−0.037	0.007	3.80E-08
rs62511803	115992376	8	A	G	0.141	−0.021	0.003	7.50E-10
rs10820625	99203606	9	C	T	0.221	−0.016	0.003	2.90E-08
rs7467480	23354940	9	A	T	0.416	−0.016	0.002	1.70E-10
rs7108077	95838005	11	G	A	0.380	−0.013	0.002	4.90E-08
rs1370059	58523077	13	G	A	0.232	0.018	0.003	1.80E-10
rs8054111	71990651	16	G	A	0.730	−0.015	0.003	1.20E-08
rs3785354	28550667	16	T	C	0.368	0.014	0.002	3.70E-08
rs11663824	50745236	18	A	C	0.397	0.014	0.002	2.50E-08

EA, effect allele; EAF, effect allele frequency; OA, other allele; SE, standard error; SNP, single nucleotide polymorphism.

**Table 2:** Significant SNPs associated with job involves mainly walking or standing (*r*^2^ < 0.001).

**SNP**	**Position**	**Chromosome**	**EA**	**OA**	**EAF**	**Beta**	**SE**	***P***
rs11264886	153997346	1	A	G	0.283	0.019	0.003	1.10E-08
rs10922907	91193049	1	T	A	0.549	−0.019	0.003	4.50E-10
rs13019832	60710571	2	A	G	0.416	−0.019	0.003	1.50E-09
rs9836291	49697459	3	A	G	0.294	−0.027	0.003	3.30E-16
rs7661349	106066982	4	C	T	0.634	−0.020	0.003	1.60E-10
rs6882046	87968864	5	G	A	0.268	−0.025	0.003	4.90E-13
rs79248502	111012600	5	G	C	0.060	−0.038	0.006	3.10E-09
rs9341742	79440229	6	T	C	0.399	0.017	0.003	2.00E-08
rs1487445	98565211	6	T	C	0.482	−0.031	0.003	6.80E-24
rs4731992	133702097	7	G	A	0.782	−0.022	0.004	1.30E-09
rs78928669	71782460	7	G	A	0.034	−0.048	0.008	7.70E-09
rs603625	95554283	11	A	G	0.419	−0.018	0.003	2.20E-09
rs6603030	83230103	15	G	A	0.794	−0.021	0.004	2.40E-08
rs3785354	28550667	16	T	C	0.368	0.018	0.003	7.50E-09
rs8054111	71990651	16	G	A	0.730	−0.021	0.003	5.30E-10
rs613872	53210302	18	T	G	0.825	0.024	0.004	3.90E-09

EA, effect allele; EAF, effect allele frequency; OA, other allele; SE, standard error; SNP, single nucleotide polymorphism.

### Statistical analysis

As the primary analysis approach, inverse variance weighted (IVW) MR was employed (18). This method operates under the assumption that the instrumental variables employed are valid and the average pleiotropic effect is null. However, bias can arise from horizontal pleiotropy when genetic instruments influence the outcome through pathways unrelated to the exposure. To account for potential pleiotropy, we supplemented the IVW method with sensitivity analysis including maximum likelihood, Mendelian randomization-Egger (MR-Egger), and weighted median (18–20). In maximum likelihood-based MR, the genetic impacts on exposures and outcomes are directly modeled as a bivariate normal distribution through the implementation of a maximum likelihood approach. Employing the MR-Egger method allows for the examination of genetic variants' pleiotropic impact on the outcome (19). Additionally, MR-Egger generates a consistent estimation of the causal association, relying on the assumption of INstrument Strength Independent of Direct Effect (InSIDE) (19). For the weighted median method to be applicable, it is necessary for valid genetic variants to represent at least 50% of the total weight of the instrument (20). Moreover, we conducted leave-one-SNP-out analyses to check whether any specific SNP drove the causal relationship, and this could evaluate the robustness of the IVW estimates. The potential impact of pleiotropy was assessed by analyzing the regression intercept obtained through the MR-Egger method (19). In addition, the MR-PRESSO method includes a global significance test for the detection of horizontal pleiotropy (21–23). The application of Cochran's Q statistic allowed for the evaluation of heterogeneity in SNP estimates within each MR association. An online tool was used to evaluate statistical power (24). The results of the statistical power calculation are presented in Supplementary Table 2. All tests were conducted with two-sided analyses using the TwoSampleMR and MR-PRESSO packages in R software (version 4.1.3). The main manuscript and Supplementary Tables 1, 2 contain all the data and results generated in this study.

## Results

**Figure 2:**
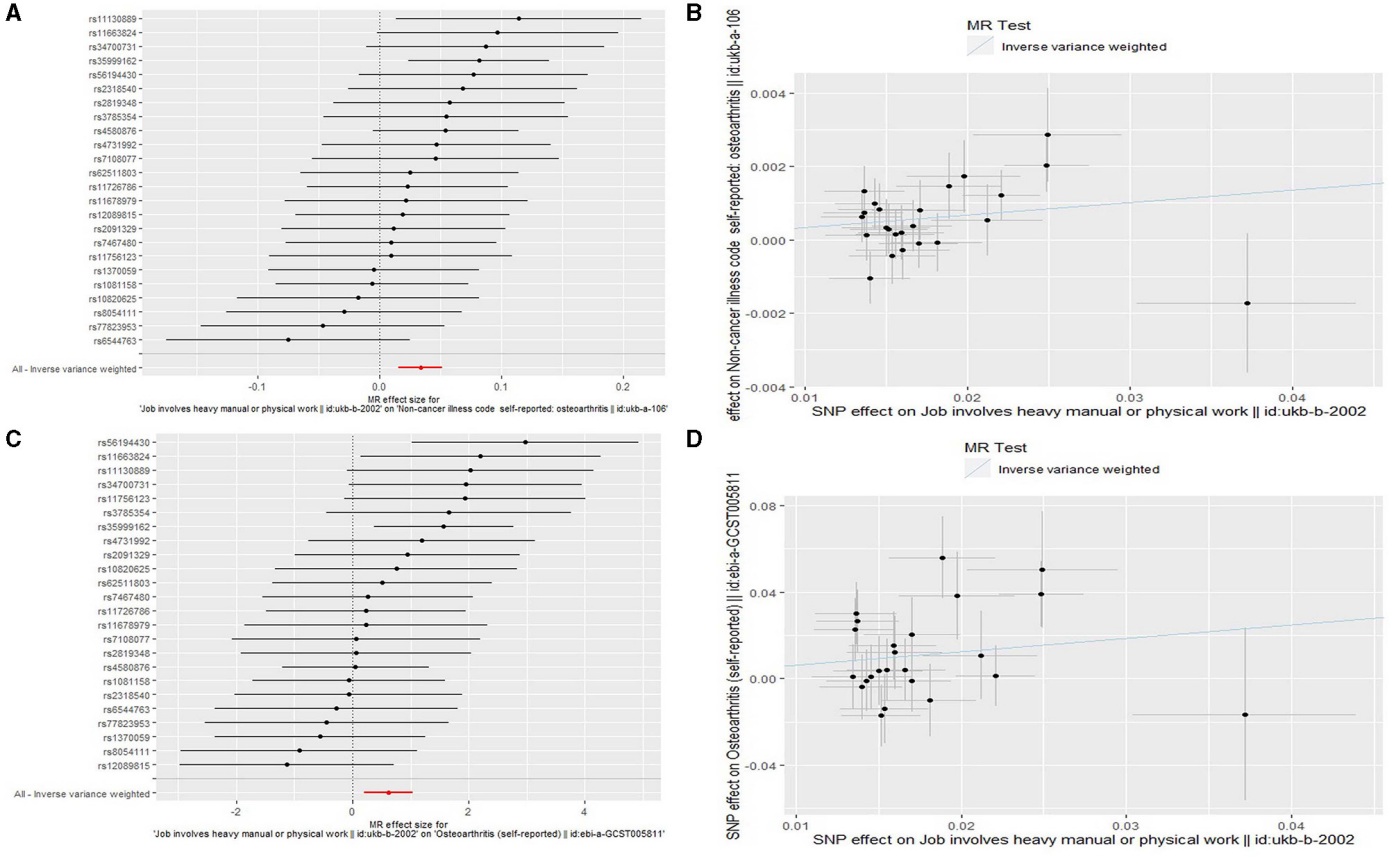
Forest plots and scatter plots for the Mendelian randomization (MR) analyses analyzing the causal association of job involves heavy manual or physical work with osteoarthritis. **(A)** A forest plot for the discovery MR analysis analyzing the causal association of job involves heavy manual or physical work with osteoarthritis. **(B)** A scatter plot for the discovery MR analysis analyzing the causal association of job involves heavy manual or physical work with osteoarthritis. **(C)** A forest plot for the replication MR analysis analyzing the causal association of job involves heavy manual or physical work with osteoarthritis. **(D)** A scatter plot for the replication MR analysis analyzing the causal association of job involves heavy manual or physical work with osteoarthritis.

**Figure 3:**
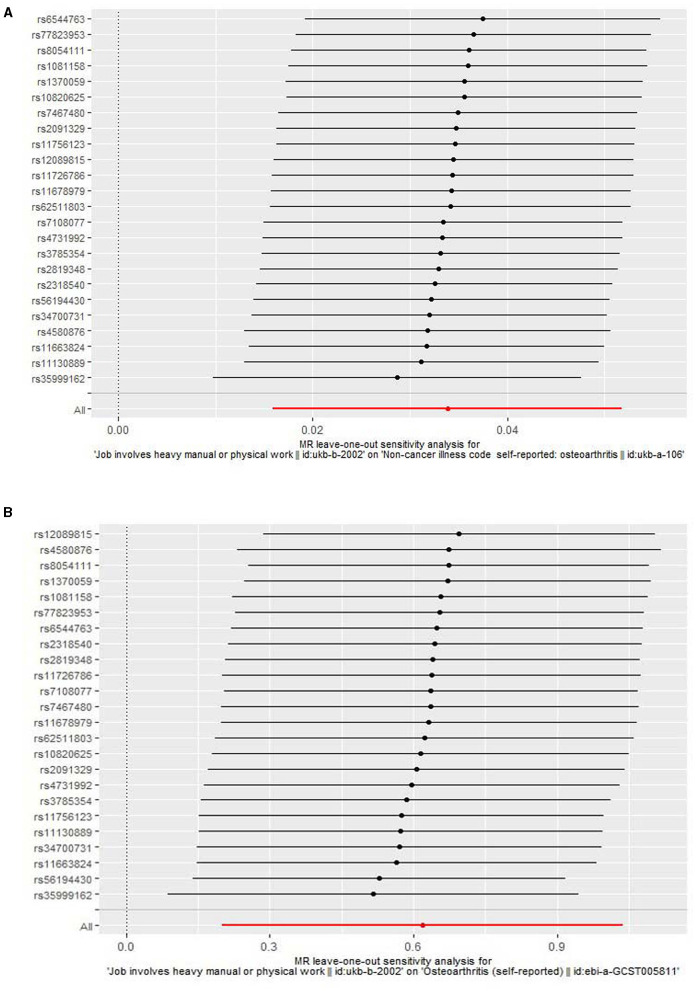
A leave-one-out analysis for the association of job involves heavy manual or physical work with osteoarthritis. **(A)** A leave-one-out analysis using the discovery outcome dataset. **(B)** A leave-one-out analysis using the replication outcome dataset.

We found that genetically predicted job involves heavy manual or physical work was significantly associated with OA in both the discovery [odds ratio (OR) = 1.034, 95% confidence interval (CI): 1.016–1.053, *P* = 2.257 × 10^−4^] and replication (OR = 1.857, 95% CI: 1.223–2.822, *P* = 0.004) analyses (Table 3 and Figure 2). The observed association was confirmed to be reliable through a heterogeneity check, as there was no heterogeneity found in the distribution of effect estimates of individual SNPs (Cochran's *Q*-test *P* = 0.486 for the discovery analysis; Cochran's *Q*-test *P* = 0.200 for the replication analysis) (Table 4). The causality between genetically predicted job involves heavy manual or physical work and OA was supported by sensitivity analyses including weight median, maximum likelihood, and MR-PRESSO (Table 3). Directional pleiotropy was not observed (MR-Egger intercept *P* = 0.316 for the discovery analysis and *P* = 0.524 for the replication analysis) (Table 4). The global test of the MR-PRESSO approach did not identify horizontal pleiotropy (Table 4). The leave-one-out analysis demonstrated that the removal of a single SNP did not substantially change the effect estimates (Figure 3).

**Table 3:** Causal effect estimates on the association between occupational factors and OA and RA.

**Stage**	**Occupational factor**	**Outcome**	**MR method**	**Number of SNPs**	**Causal effect estimate**		
					**OR**	**95% CI**	***P*-value**
Discovery	Job involves heavy manual or physical work	OA	IVW	24	1.034	1.016–1.053	2.257 × 10^−4^
			Weighted median	24	1.027	1.001–1.054	0.043
			Maximum likelihood	24	1.035	1.016–1.054	2.494 × 10^−4^
			MR-PRESSO	24	1.034	1.017–1.050	0.001
			MR-Egger	24	1.081	0.992–1.178	0.086
	Job involves heavy manual or physical work	RA	IVW	24	1.003	0.999–1.007	0.159
		Weighted median	24	1.005	0.999–1.011	0.141
		Maximum likelihood	24	1.003	0.999–1.007	0.153
		MR-PRESSO	24	1.003	0.999–1.005	0.157
		MR-Egger	24	1.012	0.992–1.032	0.261
	Job involves mainly walking or standing	OA	IVW	15	1.015	0.994–1.036	0.156
			Weighted median	15	1.014	0.989–1.039	0.283
			Maximum likelihood	15	1.015	0.998–1.033	0.086
			MR-PRESSO	15	1.015	0.996–1.034	0.151
			MR-Egger	15	1.021	0.932–1.118	0.664
	Job involves mainly walking or standing	RA	IVW	15	0.999	0.994–1.003	0.543
		Weighted median	15	0.998	0.992–1.004	0.543
		Maximum likelihood	15	0.999	0.994–1.003	0.476
		MR-PRESSO	15	0.999	0.994–1.003	0.553
		MR-Egger	15	0.999	0.978–1.020	0.910
Replication	Job involves heavy manual or physical work	OA	IVW	24	1.857	1.223–2.822	0.004
			Weighted median	24	1.268	0.728–2.211	0.402
			Maximum likelihood	24	1.917	1.304–2.817	0.001
			MR-PRESSO	24	1.856	1.230–2.794	0.008
			MR-Egger	24	3.573	0.470–27.167	0.231
	Job involves heavy manual or physical work	RA	IVW	23	1.417	0.803–2.498	0.229
		Weighted median	23	1.169	0.666–2.054	0.587
		Maximum likelihood	23	1.457	0.995–2.135	0.053
		MR-PRESSO (corrected)	22	1.121	0.848–1.482	0.584
		MR-Egger	23	0.321	0.028–3.638	0.369
	Job involves mainly walking or standing	OA	IVW	15	1.284	0.830–1.986	0.261
			Weighted median	15	1.017	0.582–1.777	0.953
			Maximum likelihood	15	1.299	0.900–1.874	0.163
			MR-PRESSO	15	1.284	0.827–1.782	0.280
			MR-Egger	15	1.472	0.209–10.357	0.704
	Job involves mainly walking or standing	RA	IVW	15	1.241	0.834–1.845	0.287
		Weighted median	15	0.949	0.550–1.637	0.850
		Maximum likelihood	15	1.251	0.869–1.803	0.229
		MR-PRESSO	15	1.241	0.716–1.785	0.305
		MR-Egger	15	0.323	0.055–1.899	0.233

CI, confidence interval; IVW, inverse variance weighted; MR-Egger, Mendelian randomization-Egger; MR-PRESSO, Mendelian Randomization-Pleiotropy RESidual Sum and Outlier; OA, osteoarthritis; OR, odds ratio; RA, rheumatoid arthritis; SNP, single nucleotide polymorphism.

**Table 4:** Heterogeneity and pleiotropy detection.

**Stage**	**Occupational factor**	**Outcome**	**Test of heterogeneity**		**MR-Egger regression**		**MR-PRESSO global test**
			**Cochran's Q**	***P*-value**	**Intercept**	***P*-value**	***P*-value**
Discovery	Job involves heavy manual or physical work	OA	22.576	0.486	−0.001	0.316	0.539
			RA	21.414	0.556	−0.002	0.389	0.614
	Job involves mainly walking or standing	OA	19.763	0.138	−0.0001	0.899	0.185
			RA	19.277	0.155	−5.647 × 10^−6^	0.982	0.164
Replication	Job involves heavy manual or physical work	OA	28.438	0.200	−0.011	0.524	0.218
			RA	52.862	2.374 × 10^−4^	0.027	0.231	< 0.001
	Job involves mainly walking or standing	OA	20.551	0.114	−0.003	0.890	0.122
			RA	17.096	0.251	0.031	0.152	0.242

OA, osteoarthritis; RA; rheumatoid arthritis.

The IVW MR results did not show a statistically significant causal association between genetically predicted job involves heavy manual or physical work and RA (discovery dataset: OR = 1.003, 95% CI: 0.999–1.007, *P* = 0.159; replication dataset: OR = 1.417, 95% CI: 0.803–2.498, *P* = 0.229) (Table 3). Null effect estimates were also found in sensitivity analyses using the weighted median, maximum likelihood, and MR-Egger models (Table 3). Evidence of effect heterogeneity (Cochran's Q-test *P* = 2.374 × 10^−4^) was detected, but the MR-Egger intercept did not suggest directional pleiotropy (Table 4). The MR-PRESSO approach identified one SNP as an outlier (rs11678979); the outlier-corrected analysis after removing this SNP showed consistent results with the IVW estimate (OR = 1.121, 95% CI: 0.848–1.482, *P* = 0.584) (Table 3). When conducting a leave-one-out analysis, it was observed that the exclusion of any individual SNP did not lead to significant alterations in the effect estimates (not shown).

Next, we evaluated the association of genetically predicted job involves mainly walking or standing with OA and RA. No evidence of causal effects was detected in either the primary MR analysis using the IVW method or sensitivity analyses using other MR models (Table 3). There was no effect of heterogeneity and directional pleiotropy (Table 4). The global test of the MR-PRESSO approach did not indicate horizontal pleiotropy (Table 4).

## Discussion

In this large two-sample MR study using summary-level data, we found that genetically predicted job involves heavy manual or physical work was associated with an increased risk of OA but not RA in individuals of European ancestry. We did not find a significant association between genetically predicted job involves mainly walking or standing and OA and RA.

The results we obtained are in line with prior observational studies, which have consistently indicated that heavy labor jobs pose a risk for OA (5–10). Although the specific mechanisms are not entirely clear, there are potential biological explanations that have been proposed for this association. First, one possible mechanism by which job involves heavy manual or physical work may increase the risk of OA is through joint overloading. The joints can experience heightened stress and strain on the articular cartilage when subjected to repetitive or excessive loading, such as heavy lifting or carrying, which may ultimately result in cartilage degradation and the development of OA over time. Second, job involves heavy manual or physical work often involves a higher risk of joint injuries and microtrauma (25–28). Acute injuries, such as fractures or ligament tears, can disrupt normal joint mechanics and increase the likelihood of developing OA later in life. Additionally, repetitive microtrauma can lead to cumulative damage to the joint structures, triggering inflammation and cartilage degeneration (28). Third, job involves heavy manual or physical work can induce chronic low-grade inflammation in the joints (29). Inflammatory responses are known to contribute to the development and progression of OA (30). Inflammatory cytokines, such as tumor necrosis factor-α and interleukin-1β, can promote cartilage breakdown, induce synovial changes, and inhibit repair mechanisms, thereby increasing the risk of OA (31). Fourth, many jobs involving heavy physical labor often do not provide sufficient rest and recovery time for the musculoskeletal system (25). The absence of adequate rest intervals during continuous exertion may impede the proper repair and reconstruction of tissues, which can negatively affect the health of joint structures, increasing their susceptibility to degeneration and OA. Fifth, the risk of OA in heavy physically demanding occupations can be significantly influenced by ergonomic factors (32, 33). Improper joint alignment, excessive joint loading, and unnatural movement patterns can result from poorly designed workstations or equipment (32, 33). These factors may contribute to joint misalignment and heighten the likelihood of cartilage wear and tear, thus promoting the development of OA.

Our study highlights the need for stringent occupational health and safety regulations to protect workers engaged in heavy manual or physically demanding jobs. Employers should implement appropriate measures to minimize the risk of developing OA among their employees, such as providing adequate rest breaks, ergonomic equipment, proper training on work-related techniques, and early detection of OA. In addition, our results may increase public awareness of the causal relationship of job involves heavy manual or physical work with OA. Individuals should pay more attention to their occupational choices and work environments to protect their health. Furthermore, our study may provide new research directions for future MR studies on OA, such as exploring whether other occupational factors play a role in the development of OA and how to prevent and treat OA.

In agreement with a number of observational studies (5, 34–37), we found no causal association between job involves mainly walking or standing and OA. A recent comprehensive systematic review and meta-analysis summarizing 11 case–control or cohort studies also suggested no increased risk of OA related to walking and standing (38). In addition to the results related to OA, we estimated whether job involves heavy manual or physical work and job involves mainly walking or standing are associated with RA: however, we did not find a causal link. Compared with observational studies, our study has several strengths related to study design. First, using a two-sample MR approach, our analysis minimized the impact of confounding, reverse causation, and exposure measurement errors that are non-differential. Second, with a focus on robustness, our study adopted a strategy to avert weak instrument bias. This was achieved by limiting our selection to SNPs demonstrating noteworthy instrument strength (*F* > 10) and an association with their respective outcomes at the GWAS significance threshold (*P* < 5 × 10^−8^). The exclusion of SNPs in linkage disequilibrium added an additional layer of refinement to our approach. Third, despite the impossibility of entirely dismissing pleiotropy, we tackled this issue through diverse sensitivity analyses. The robustness of the MR estimates was evident across the different sensitivity analyses conducted. It is worth mentioning that, as a reliable approach for sensitivity analysis, the MR-PRESSO method can identify and exclude potential outlier SNPs that display horizontal pleiotropic effects (21). It showed homogeneous results with the IVW method in our analyses. Fourth, the validation of our findings using additional large GWAS datasets yielded comparable causal estimates.

Despite these strengths, caution should still be employed because our study has limitations. First, inflated type 1 error rates and biased effect estimates may arise if the assumption of independent samples for the instrument variable and the outcome is violated in two-sample MR. However, this consideration is only relevant when dealing with continuous outcomes, which differ from the binary outcomes investigated in our current study (39). Second, within the evidence-based pyramid, MR studies are positioned below randomized clinical trials (RCTs) and meta-analyses of RCTs. Thus, well-designed RCTs are necessary to confirm our findings in the future. Third, as our study solely involved European participants, it is important to avoid generalizing the findings to diverse ethnic populations. However, there are several advantages to using summary statistics from a single ethnic population. For example, it ensures a more homogeneous genetic background, reducing confounding due to population stratification and minimizing bias in the causal estimates. In addition, European populations often have a more extensive collection of genome-wide association studies, providing a larger sample size and greater statistical power for analysis. In anticipation of the availability of extensive GWAS summary-level data on occupational factors in various ethnic groups including East Asians and Latin Americans, it is our hope that future MR studies can encompass a greater range of ethnic groups, which would enable the exploration of the causal link between the two occupational factors and OA and RA in a more diverse population. Fourth, although our MR study evaluated causality for association, it could not assess whether intervening in the two occupational factors would have beneficial effects for OA and RA. We anticipate that future meticulously planned RCTs would assess the positive impact of intervening in these occupational factors on diminishing the likelihood of OA and RA.

In conclusion, our large two-sample MR study demonstrated that genetically predicted job involves heavy manual or physical work was causally associated with OA but not RA risk. Our data provided no evidence that genetically predicted job involves mainly walking or standing was related to OA and RA. Our findings may help in developing strategies to prevent OA by focusing on limiting the amount and duration of physically demanding job-related activities.

## Data availability statement

Analysis in this study was conducted using datasets that are publicly available. These datasets can be accessed through the IEU Open GWAS Project database at https://gwas.mrcieu.ac.uk/.

## Author contributions

JH: Conceptualization, Data curation, Formal analysis, Investigation, Methodology, Resources, Software, Validation, Visualization, Writing – original draft, Writing – review & editing.
