# Supplementary material for: The causal effect of two occupational factors on osteoarthritis and rheumatoid arthritis: a Mendelian randomization study
Source: Front Public Health. 2024 Feb 12;11:1281214. doi: 10.3389/fpubh.2023.1281214 (PMC10895025; doi:10.3389/fpubh.2023.1281214)
Supplement: Supplementary file 2 [file Table_2.DOCX]

Supplementary Table 2 Power calculation.

| Stage | Occupational factor | Outcome | OR ≤ | OR ≥ |
| --- | --- | --- | --- | --- |
| Discovery | job involves heavy manual or physical work | OA | 0.68 | 1.34 |
|  |  | RA | 0.63 | 2.33 |
|  | job involves mainly walking or standing | OA | 0.70 | 1.32 |
|  |  | RA | 0.57 | 2.26 |
| Replication | job involves heavy manual or physical work | OA | 0.49 | 1.65 |
|  |  | RA | 0.56 | 1.57 |
|  | job involves mainly walking or standing | OA | 0.51 | 1.61 |
|  |  | RA | 0.58 | 1.53 |

ORs were calculated by setting 80% of power.
